# The crystal structure, Hirshfeld surface analysis and energy frameworks of 2-[2-(meth­oxy­carbon­yl)-3,6-bis­(meth­oxy­meth­oxy)phen­yl]acetic acid

**DOI:** 10.1107/S2056989020007987

**Published:** 2020-06-19

**Authors:** Mustapha Tiouabi, Raphaël Tabacchi, Helen Stoeckli-Evans

**Affiliations:** a Institute of Chemistry, University of Neuchâtel, Av. de Bellevax 51, CH-2000 Neuchâtel, Switzerland; b Institute of Physics, University of Neuchâtel, rue Emile-Argand 11, CH-2000 Neuchâtel, Switzerland

**Keywords:** crystal structure, isocoumarin, hydrogen bonding, C—H⋯π inter­actions, offset π–π inter­actions, supra­molecular framework, Hirshfeld surface analysis, energy frameworks

## Abstract

In the title compound, C_14_H_18_O_8_, (**I**), the meth­oxy­carbonyl [–C(=O)OCH_3_] and the acetic acid [–CH_2_C(=O)OH] groups are inclined to the benzene ring by 79.24 (11) and 76.71 (13)°, respectively, and are normal to each other with a dihedral angle of 90.00 (13)°. In the crystal, mol­ecules are linked by a pair of O—H⋯O hydrogen bonds forming the familiar acetic acid inversion dimer. The dimers are linked by two C—H⋯O hydrogen bonds and an offset π–π inter­action [inter­centroid distance = 3.6405 (14) Å], forming layers lying parallel to the (10) plane. The layers are linked by a third C—H⋯O hydrogen bond and a C—H⋯π inter­action to form a supra­molecular framework.

## Abstract 2

The title compound, 2-(2-(meth­oxy­carbon­yl)-3,6-bis­(meth­oxy­meth­oxy)phen­yl)acetic acid, was synthesized as an inter­mediate for a possible total synthesis of the isocoumarin 3-methyl-3,5,8-trihy­droxy-3,4-di­hydro­isocoumarin.

## Chemical context   

Isocoumarins are among the phytotoxins produced by the *Ceratocystis fimbriata* species. The latter are pathogenic agents responsible for the infections of coffee and plane trees (Gremaud & Tabacchi, 1994 ▸; Bürki *et al.*, 2003 ▸). The analysis of the culture medium of *Ophiostoma ulmi*, a pathogenic agent responsible for elm disease and classified in the family of *Ceratocystis*, enabled Michel (2001 ▸) to isolate sixteen metabolites including four isocoumarins without apparent toxicity and a new natural product, 3-methyl-3,5,8-trihy­droxy-3,4-di­hydro­isocoumarin, found in the extract of diseased wood. Qualitatively, the latter is present in trace amounts; however, the toxicity of this metabolite is possible, since the activity is not necessarily proportional to the concentration.
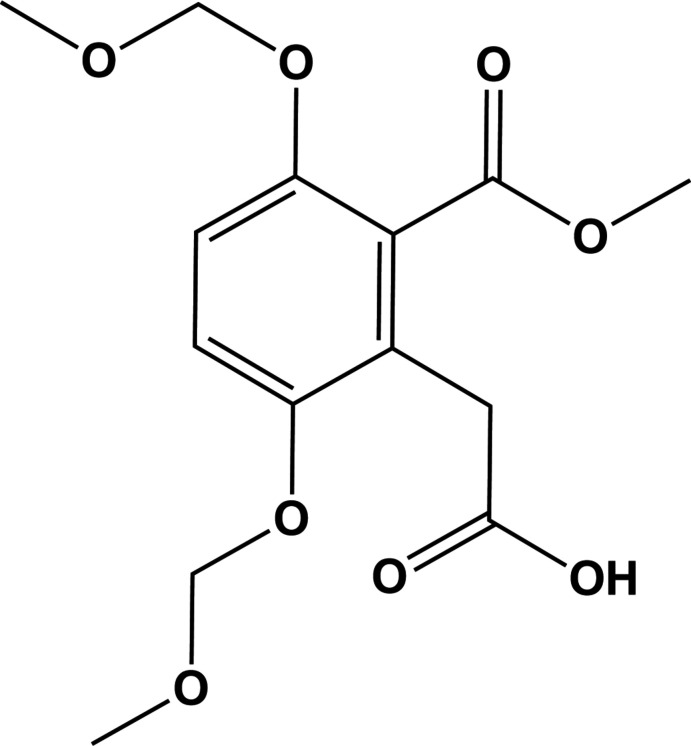


**Figure 1:**
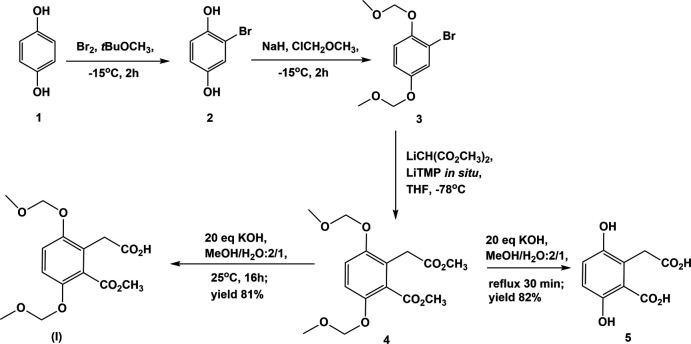
The reaction scheme resulting in the formation of the title compound, **I**.

The title compound (**I**), is a key inter­mediate for the proposed total synthesis of 3-methyl-3,5,8-trihy­droxy-3,4-di­hydro­isocoumarin, and its synthesis is illustrated in Fig. 1 ▸ (Tiouabi, 2005 ▸). It was synthesized from hydro­quinone (**1**), which was first brominated to give compound **2**. The latter was then reacted with NaH and ClCH_2_OCH_3_ to give compound **3**, so protecting the hydroxyl groups. Reacting **3** with tetra­methyl­piper­idene with *n*-butyl­lithium and CH_2_(CO_2_CH_3_)_2_ resulted in the formation of compound **4**. Finally **4** was reacted with various qu­anti­ties of KOH in methanol/water (2:1) to give the title compound, **I**. The highest yield (81%) was obtained by reacting 20 equivalents of KOH in methanol/water (2:1) at 298 K under stirring for 16 h. Inter­estingly, the same reaction with reflux for 30 minutes yielded the diacid, 2-(carb­oxy­meth­yl)-3,6-di­hydroxy­benzoic acid (**5**), with a yield of 82% (Fig. 1 ▸).

## Structural commentary   

**Figure 2:**
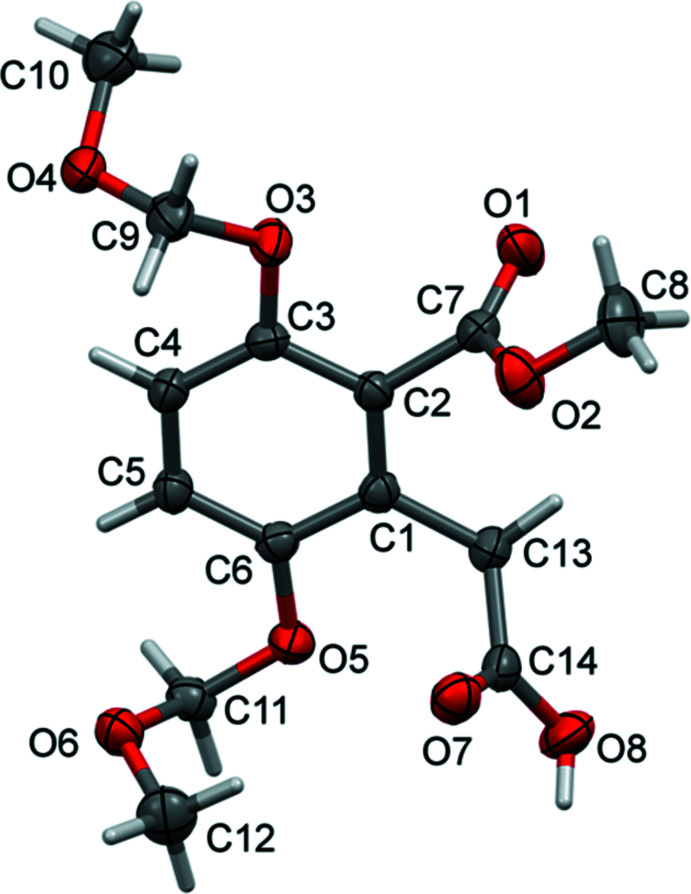
The mol­ecular structure of compound **I**, with atom labelling. Displacement ellipsoids are drawn at the 50% probability level.

The mol­ecular structure of compound **I** is illustrated in Fig. 2 ▸. The meth­oxy­methyl group (mean plane 1: C2/C7/O1/O2/C8; r.m.s. deviation = 0.009 Å) is inclined to the benzene ring by 79.24 (11)°. The plane of the acetic acid unit (mean plane 2: C13/C14/O7/O8; r.m.s. deviation = 0.014 Å) is inclined to the benzene ring by 76.71 (13) °. Planes 1 and 2 are normal to each other with a dihedral angle of 90.00 (13)°. The meth­oxy­meth­oxy side chains (O3–C9–O4–C10 and O5–C11–O6–C12) are displaced to opposite sides of the benzene ring. They have twisted conformations as seen from the torsion angles given in Table 3 ▸.

## Supra­molecular features   

**Figure 3:**
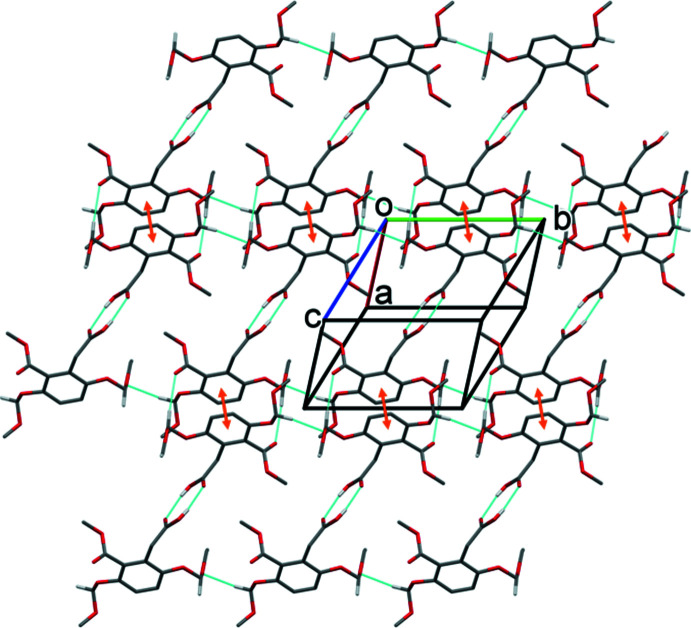
A view normal to plane (10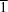) of the layer structure in the crystal of compound **I**. Hydrogen bonds (Table 1 ▸) are shown as dashed lines and offset π–π inter­actions as orange double arrows. For clarity, only the H atoms involved in the inter­molecular inter­actions have been included.

**Figure 4:**
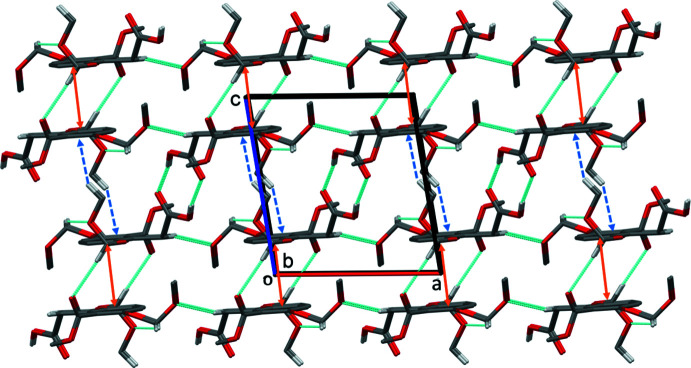
A view along the *b* axis of the crystal packing of compound **I**. The hydrogen bonds (Table 1 ▸) are shown as dashed lines. The offset π–π inter­actions are indicated by orange double arrows, and the C—H⋯π inter­actions by blue dashed arrows. For clarity, only the H atoms involved in the inter­molecular inter­actions have been included.

In the crystal of **I**, mol­ecules are linked by a pair of O—H⋯O hydrogen bonds (O8—H8⋯O7^i^) forming an inversion dimer with an 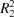
(8) ring motif (Fig. 3 ▸ and Table 1 ▸). The dimers are linked by two C—H⋯O hydrogen bonds (C9—H9*B*⋯O6^ii^ and C11—H11*A*⋯O1^iii^) and offset π–π inter­actions between inversion-related benzene rings, so forming layers lying parallel to (10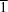). The layers are linked by a third C—H⋯O hydrogen bond (C13—H13*B*⋯O4^iv^) and a C—H⋯π inter­action to form a supra­molecular framework (Table 1 ▸ and Fig. 4 ▸). Details of the offset π–π inter­action are as follows: *Cg*⋯*Cg* ^iii^ = 3.6405 (14) Å, where *Cg* is the centroid of the C1–C6 benzene ring; inter­planar distance = 3.5911 (9) Å; offset = 0.597 Å; symmetry code: (iii) −*x*, −*y* + 1, −*z*.

## Hirshfeld surface analysis and two-dimensional fingerprint plots   

The Hirshfeld surface analysis (Spackman & Jayatilaka, 2009 ▸), the associated two-dimensional fingerprint plots and the calculation of the energy frameworks (McKinnon *et al.*, 2007 ▸) were performed with *CrystalExplorer17.5* (Turner *et al.*, 2017 ▸), following the protocol outlined in the recent article by Tiekink and collaborators (Tan *et al.*, 2019 ▸). The Hirshfeld surface is colour-mapped with the normalized contact distance, *d*_norm_, from red (distances shorter than the sum of the van der Waals radii) through white to blue (distances longer than the sum of the van der Waals radii). The energy frameworks (Turner *et al.*, 2015 ▸; Tan *et al.*, 2019 ▸) are represented by cylinders joining the centroids of mol­ecular pairs using red, green and blue colour codes for the electrostatic (*E*_ele_), dispersion (*E*_dis_) and total energy (*E*_tot_) components, respectively. The radius of the cylinder is proportional to the magnitude of the inter­action energy.

**Figure 5:**
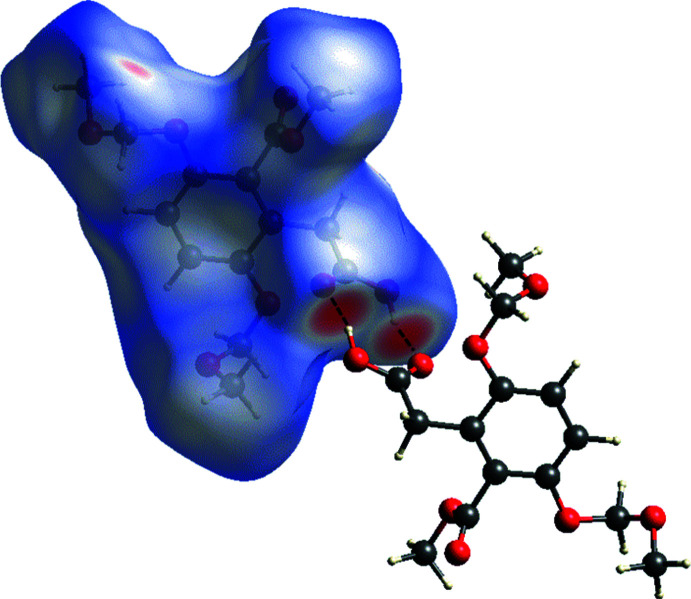
The Hirshfeld surface of compound **I** mapped over *d*_norm_, in the colour range −0.6996 to 1.3669 a.u..

**Figure 6:**
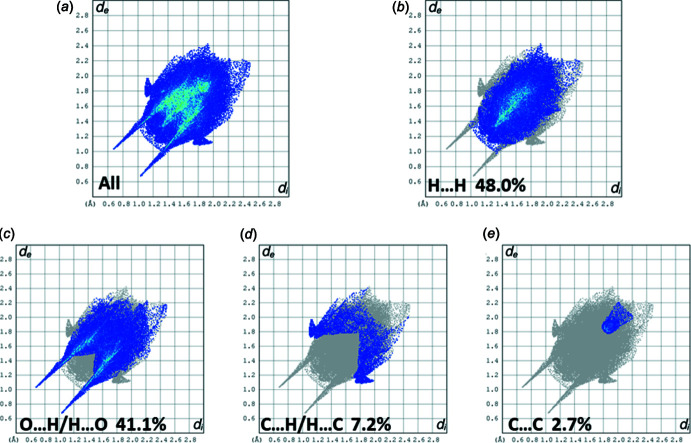
(*a*) The full two-dimensional fingerprint plot for compound **I**, and fingerprint plots delineated into (*b*) H⋯H (48.0%), (*c*) O⋯H/H⋯O (41.1%), (*d*) C⋯H/H⋯C (7.2%) and (*e*) C⋯C (2.7%) contacts.

**Figure 7:**
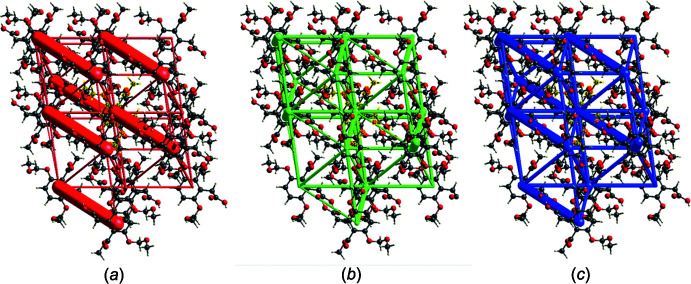
The energy frameworks for **I** viewed down the *c*-axis direction comprising, (*a*) electrostatic potential forces (*E*_ele_), (*b*) dispersion forces (*E*_dis_) and (*c*) total (*E*_tot_) energy for a cluster about a reference mol­ecule of **I**. The energy frameworks were adjusted to the same scale factor of 80 with a cut-off value of 5 kJ mol^−1^ within a 5 Å radius of a selected central mol­ecule.

A view of the Hirshfeld surface of **I** mapped over *d*_norm_ is shown in Fig. 5 ▸. The short inter­atomic O⋯H/H⋯O contacts are indicated by the large red spots. Other C—H⋯O contacts are indicated by faint red spots. A full list of short inter­atomic contacts in the crystal of **I** are given in Table 2 ▸. The majority of the significant contacts are O⋯H and C⋯H contacts, as confirmed by the two-dimensional fingerprint plots (Fig. 6 ▸). The principal inter­molecular contacts for **I** are delineated into H⋯H (48.0%) (Fig. 6 ▸ *b*), O⋯H/H⋯O (41.1%) (Fig. 6 ▸ *c*), C⋯H/H⋯C (7.2%) (Fig. 6 ▸ *d*) and C⋯C (2.7%) (Fig. 6 ▸ *e*) contacts. The inter­molecular contacts are therefore almost equally distributed between electrostatic and dispersion forces, as shown in Fig. 7 ▸ *a* and 7*b*. The energy frameworks (Fig. 7 ▸) were adjusted to the same scale factor of 80 with a cut-off value of 5 kJ mol^−1^ within a radius of 5 Å about a central mol­ecule, and were obtained using the wave function calculated at the HF/3-21G level of theory.

**Figure 8:**
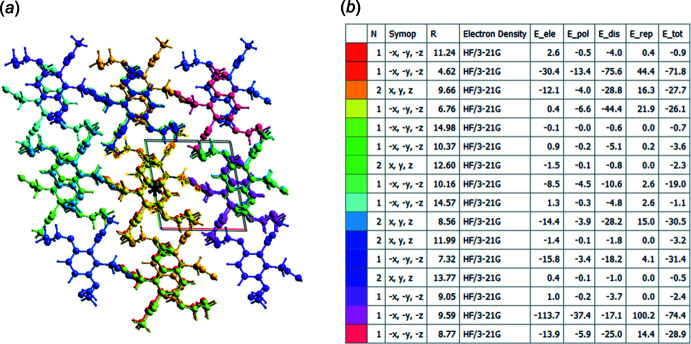
The colour-coding inter­action mapping within 5 Å of the centering (*) mol­ecular cluster.

The calculation of the energy framework results in a colour-coded mol­ecular cluster related to the specific inter­action energy, see Fig. 8 ▸ *a*. The individual energy components, electrostatic (*E*_ele_), polarization (*E*_pol_), dispersion (*E*_dis_) and repulsion (*E*_rep_) energies and the sum of these components (*E*_tot_) for the inter­actions relative to a reference mol­ecule (*) are shown in Fig. 8 ▸ *b*.

## Database survey   

**Figure 9:**
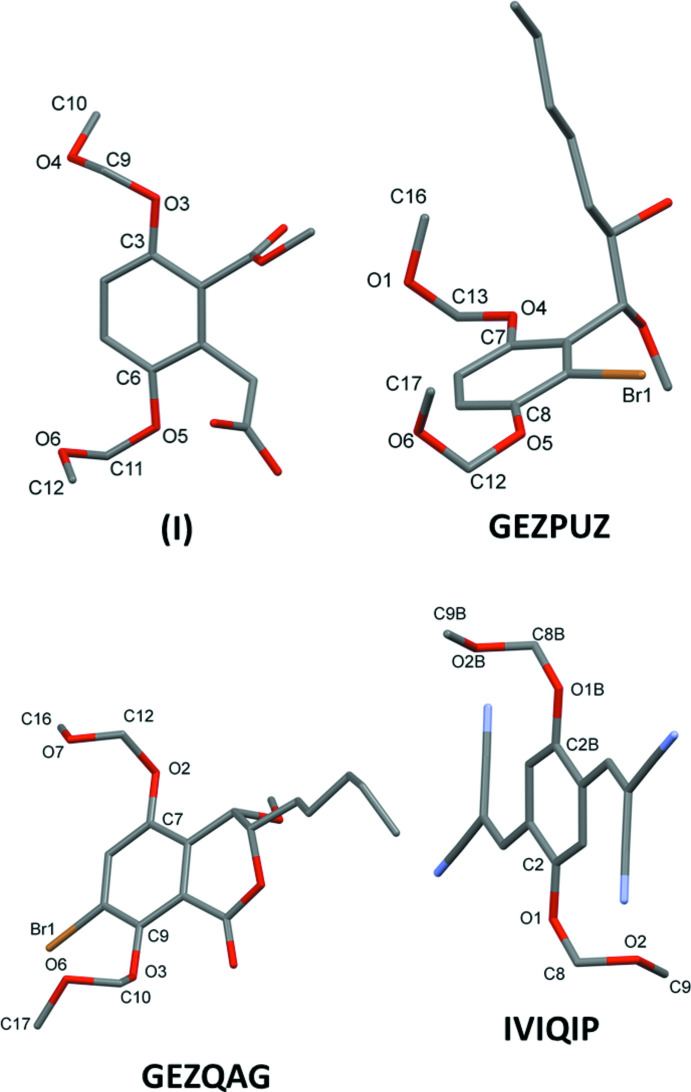
A view of the mol­ecular structures of **I**, GEZPUZ, GEZQAGr and IVIQIP. The original atom-labelling schemes have been used for the latter three compounds. For clarity, H atoms have been omitted

A search of the Cambridge Structural Database (CSD, Version 5.41, last update March 2020; Groom *et al.*, 2016 ▸) for the 3,6-bis­(meth­oxy­meth­oxy)phenyl substructure gave only six hits. Three compounds are of particular inter­est, namely 1-[2-bromo-3,6-bis­(meth­oxy­meth­oxy)phen­yl]-1-meth­oxy­hep­tan-2-ol (CSD refcode GEZPUZ; Nakayama *et al.*, 2018 ▸), 7-bromo-4-meth­oxy-5,8-bis­(meth­oxy­meth­oxy)-3-pentyl-3,4-di­hydro-1*H*-2-benzo­pyran-1-one (GEZQAG; Nakayama *et al.*, 2018 ▸) and 2,2′-{[2,5-bis­(meth­oxy­meth­oxy)-1,4-phenyl­ene]di­methylyl­idene}dimalono­nitrile (IVIQIP; Zhang *et al.*, 2017 ▸). The first two, GEZPUZ and GEZQAG [compounds 17 and 20 in the publication by Nakayama *et al.* (2018 ▸)], are key inter­mediates in the synthesis of the di­hydro­isocoumarin-type natural products, eurotiumide A and eurotiumide B. Compound IVIQIP [compound 1c in the publication by Zhang *et al.* (2017 ▸)] was synthesized in a study of organic solid fluoro­phores. The conformation of the –O–CH_2_–O–CH_3_ side chains are compared to that in compound **I** in Fig. 9 ▸ and Table 3 ▸. In GEZPUZ and GEZQAG these side chains are twisted and directed to the same side of the benzene ring. In IVIQIP they are also twisted but directed to opposite sides of the benzene ring as in compound **I**.

A search of the CSD for the substructure 2-(2-(meth­oxy­carbon­yl)phen­yl)acetic acid gave zero hits.

## Synthesis and crystallization   

The synthesis of compound **I** is illustrated in Fig. 1 ▸. Full details of the syntheses and spectroscopic and analytical data for compounds **2**–**5** and **I** are available in the PhD thesis of Tiouabi (2005 ▸). It can be downloaded from the website https://doc.rero.ch/record, a digital library where many theses of Swiss universities are deposited. Colourless block-like crystals of **I** were obtained by slow evaporation of a solution in acetone-*d*_6_.

## Refinement   

Crystal data, data collection and structure refinement details are summarized in Table 4 ▸. The OH and C-bound H atoms were included in calculated positions and treated as riding atoms: O—H = 0.84 Å, C—H = 0.95–0.99 Å with *U*_iso_(H) = 1.5*U*_eq_(OH and C-meth­yl) and 1.2*U*_eq_(C) for other H-atoms.

Intensity data were measured using a Stoe IPDS I, a one-circle diffractometer. For the triclinic system often only 93% of the Ewald sphere is accessible, which explains why the alert diffrn_reflns_laue_measured_fraction_full value (0.942) below minimum (0.95) is given. This involves 155 random reflections out of the expected 2692 for the IUCr cutoff limit of sin θ/λ = 0.60.

## Supplementary Material

Crystal structure: contains datablock(s) I, Global. DOI: 10.1107/S2056989020007987/ex2033sup1.cif

Structure factors: contains datablock(s) I. DOI: 10.1107/S2056989020007987/ex2033Isup2.hkl

Supporting information file. DOI: 10.1107/S2056989020007987/ex2033Isup3.cml

CCDC reference: 2009890

Additional supporting information:  crystallographic information; 3D view; checkCIF report

## Figures and Tables

**Table 1:** Hydrogen-bond geometry (Å, °) *Cg* is the centroid of the C1–C6 benzene ring.

*D*—H⋯*A*	*D*—H	H⋯*A*	*D*⋯*A*	*D*—H⋯*A*
O8—H8⋯O7^i^	0.84	1.85	2.676 (2)	168
C9—H9*B*⋯O6^ii^	0.99	2.43	3.256 (3)	140
C11—H11*A*⋯O1^iii^	0.99	2.38	3.366 (3)	175
C13—H13*B*⋯O4^iv^	0.99	2.50	3.421 (3)	155
C12—H12*B*⋯*Cg* ^v^	0.98	2.66	3.451 (3)	138

Symmetry codes: (i) 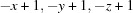; (ii) 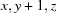; (iii) 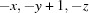; (iv) 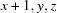; (v) 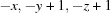.

**Table 2:** Short inter­atomic contacts (Å)^*a*^ in the crystal of compound **I**

Atom1	Atom2	Length	Length − VdW
O7	H8^i^	1.850	−0.870
O7	O8^i^	2.676	−0.364
O1	H11*A* ^iii^	2.379	−0.341
O6	H9*B* ^vi^	2.432	−0.288
O4	H13*B* ^v^	2.501	−0.219
H8	C14^i^	2.703	−0.197
O5	H10*C* ^iii^	2.637	−0.083
C12	H9*B* ^vi^	2.882	−0.018
O4	H8*A* ^v^	2.723	0.003
O7	C8^viii^	3.223	0.003
O7	H8*B* ^viii^	2.726	0.006
O2	H8*B* ^viii^	2.731	0.011
H8	H8^i^	2.416	0.016
C2	H12*B* ^vii^	2.932	0.032
O6	C9^vi^	3.256	0.036
H8*B*	C14^viii^	2.954	0.054
C9	H8*A* ^v^	2.957	0.057
C1	H12*B* ^vii^	2.968	0.068
C3	H12*B* ^vii^	2.968	0.068
H10*C*	C11^iii^	2.969	0.069
H9*B*	H8*A* ^v^	2.482	0.082

(*a*) Calculated using *Mercury* (Macrae *et al.*, 2020 ▸). Symmetry codes: (i) −*x* + 1, −*y* + 1, −*z* + 1; (iii) −*x*, −*y* + 1, −*z*; (v) *x* − 1, *y*, *z*; (vi) *x*, *y* − 1, *z*; (vii) −*x*, −*y* + 1, −*z* + 1; (viii) −*x* + 1, −*y* + 2, −*z* + 1.

**Table 3:** Selected torsion angles (°) in compound **I** compared to those in compounds GEZPUZ, GEZQAG and IVIQIP

**I**	
C3—O3—C9—O4	−77.8 (2)
C10—O4—C9—O3	−67.6 (2)
C6—O5—C11—O6	−61.3 (2)
C12—O6—C11—O5	−65.5 (2)
	
GEZPU*Z* ^*a*^	
C7—O4—C13—O1	67.1 (3)
C16—O1—C13—O4	56.3 (3)
C8—O5—C12—O6	−80.1 (2)
C17—O6—C12—O5	−65.8 (3)
	
GEZQAG^*a*^	
C7—O2—C12—O7	−71.2 (8)
C16—O7—C12—O2	−67.6 (9)
C9—O3—C10—O6	86.1 (7)
C17—O6—C10—O3	76.6 (8)
	
IVIQIP^*b*^,^*c*^	
C1—O1—C8—O2	−68.9 (2)
C9—O2—C8—O1	−66.1 (2)

(*a*) Nakayama *et al.* (2018 ▸); (*b*) Zhang *et al.* (2017 ▸); (*c*) compound IVIQIP possesses inversion symmetry.

**Table 4:** Experimental details

Crystal data	
Chemical formula	C_14_H_18_O_8_
*M*_r_	314.28
Crystal system, space group	Triclinic, *P* 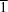
Temperature (K)	173
*a*, *b*, *c* (Å)	8.5628 (12), 9.6623 (13), 9.9767 (12)
α, β, γ (°)	112.534 (14), 94.744 (15), 97.999 (16)
*V* (Å^3^)	746.60 (19)
*Z*	2
Radiation type	Mo *K*α
μ (mm^−1^)	0.12
Crystal size (mm)	0.30 × 0.30 × 0.20

Data collection	
Diffractometer	Stoe IPDS 1
Absorption correction	Multi-scan (*MULABS*; Spek, 2020 ▸)
*T*_min_, *T*_max_	0.827, 1.000
No. of measured, independent and observed [*I* > 2σ(*I*)] reflections	6011, 2752, 1557
*R*_int_	0.065
(sin θ/λ)_max_ (Å^−1^)	0.617

Refinement	
*R*[*F* ^2^ > 2σ(*F* ^2^)], *wR*(*F* ^2^), *S*	0.045, 0.108, 0.83
No. of reflections	2752
No. of parameters	204
H-atom treatment	H-atom parameters constrained
Δρ_max_, Δρ_min_ (e Å^−3^)	0.25, −0.23

Computer programs: *EXPOSE*, *CELL* and *INTEGRATE* in *IPDS-I* (Stoe & Cie, 2004 ▸), *SHELXS97* (Sheldrick, 2008 ▸), *Mercury* (Macrae *et al.*, 2020 ▸), *SHELXL2018/3* (Sheldrick, 2015 ▸), *PLATON* (Spek, 2020 ▸) and *publCIF* (Westrip, 2010 ▸).

## supplementary crystallographic information

### Crystal data

**Table:**

C_14_H_18_O_8_	*Z* = 2
*M**_r_* = 314.28	*F*(000) = 332
Triclinic, *P*1	*D*_x_ = 1.398 Mg m^−^^3^
*a* = 8.5628 (12) Å	Mo *K*α radiation, λ = 0.71073 Å
*b* = 9.6623 (13) Å	Cell parameters from 3063 reflections
*c* = 9.9767 (12) Å	θ = 2.2–25.8°
α = 112.534 (14)°	µ = 0.12 mm^−^^1^
β = 94.744 (15)°	*T* = 173 K
γ = 97.999 (16)°	Block, colourless
*V* = 746.60 (19) Å^3^	0.30 × 0.30 × 0.20 mm

### Data collection

**Table:**

Stoe IPDS 1 diffractometer	2752 independent reflections
Radiation source: fine-focus sealed tube	1557 reflections with *I* > 2σ(*I*)
Plane graphite monochromator	*R*_int_ = 0.065
φ rotation scans	θ_max_ = 26.0°, θ_min_ = 2.3°
Absorption correction: multi-scan (MULABS; Spek, 2020)	*h* = −10→10
*T*_min_ = 0.827, *T*_max_ = 1.000	*k* = −11→11
6011 measured reflections	*l* = −11→11

### Refinement

**Table:**

Refinement on *F*^2^	Secondary atom site location: difference Fourier map
Least-squares matrix: full	Hydrogen site location: inferred from neighbouring sites
*R*[*F*^2^ > 2σ(*F*^2^)] = 0.045	H-atom parameters constrained
*wR*(*F*^2^) = 0.108	*w* = 1/[σ^2^(*F*_o_^2^) + (0.0527*P*)^2^] where *P* = (*F*_o_^2^ + 2*F*_c_^2^)/3
*S* = 0.83	(Δ/σ)_max_ < 0.001
2752 reflections	Δρ_max_ = 0.25 e Å^−^^3^
204 parameters	Δρ_min_ = −0.23 e Å^−^^3^
0 restraints	Extinction correction: (*SHELXL2018/3*; Sheldrick, 2015), Fc^*^=kFc[1+0.001xFc^2^λ^3^/sin(2θ)]^-1/4^
Primary atom site location: structure-invariant direct methods	Extinction coefficient: 0.019 (5)

### Special details

**Table:**

Geometry. All esds (except the esd in the dihedral angle between two l.s. planes) are estimated using the full covariance matrix. The cell esds are taken into account individually in the estimation of esds in distances, angles and torsion angles; correlations between esds in cell parameters are only used when they are defined by crystal symmetry. An approximate (isotropic) treatment of cell esds is used for estimating esds involving l.s. planes.

### Fractional atomic coordinates and isotropic or equivalent isotropic displacement parameters (Å^2^)

**Table:**

	*x*	*y*	*z*	*U*_iso_*/*U*_eq_	
O1	0.25754 (19)	0.8960 (2)	0.1239 (2)	0.0457 (5)	
O2	0.30589 (19)	0.94409 (18)	0.3608 (2)	0.0427 (5)	
O3	−0.08148 (17)	0.86702 (16)	0.21564 (19)	0.0354 (4)	
O4	−0.36196 (18)	0.82093 (18)	0.1605 (2)	0.0411 (5)	
O5	0.12039 (17)	0.34010 (16)	0.18992 (18)	0.0313 (4)	
O6	−0.07328 (18)	0.23819 (17)	0.29737 (19)	0.0357 (4)	
O7	0.35707 (18)	0.58696 (18)	0.45712 (19)	0.0373 (4)	
O8	0.52484 (19)	0.4650 (2)	0.3133 (2)	0.0449 (5)	
H8	0.548084	0.441888	0.384533	0.067*	
C1	0.1715 (2)	0.5912 (2)	0.2067 (2)	0.0255 (5)	
C2	0.1204 (2)	0.7231 (2)	0.2130 (2)	0.0255 (5)	
C3	−0.0431 (2)	0.7295 (2)	0.2044 (3)	0.0266 (5)	
C4	−0.1531 (2)	0.6025 (2)	0.1876 (3)	0.0290 (5)	
H4	−0.263625	0.606043	0.179939	0.035*	
C5	−0.1034 (2)	0.4714 (2)	0.1817 (3)	0.0292 (5)	
H5	−0.179938	0.384882	0.170246	0.035*	
C6	0.0580 (2)	0.4643 (2)	0.1925 (2)	0.0262 (5)	
C7	0.2333 (2)	0.8614 (2)	0.2243 (3)	0.0284 (5)	
C8	0.4160 (3)	1.0825 (3)	0.3806 (3)	0.0494 (7)	
H8A	0.495875	1.056174	0.314572	0.074*	
H8B	0.469429	1.131775	0.482449	0.074*	
H8C	0.356664	1.152557	0.357997	0.074*	
C9	−0.2285 (3)	0.8986 (3)	0.2679 (3)	0.0376 (6)	
H9A	−0.238127	0.869474	0.352219	0.045*	
H9B	−0.226769	1.009573	0.303253	0.045*	
C10	−0.3713 (3)	0.8721 (3)	0.0443 (3)	0.0509 (7)	
H10A	−0.362356	0.983164	0.085357	0.076*	
H10B	−0.473812	0.824240	−0.019781	0.076*	
H10C	−0.284222	0.844062	−0.012623	0.076*	
C11	0.0113 (3)	0.2090 (2)	0.1794 (3)	0.0314 (5)	
H11A	−0.065027	0.172092	0.087538	0.038*	
H11B	0.071162	0.126917	0.174037	0.038*	
C12	0.0227 (3)	0.2754 (3)	0.4335 (3)	0.0487 (7)	
H12A	0.078802	0.191711	0.426820	0.073*	
H12B	−0.044987	0.291178	0.510676	0.073*	
H12C	0.100661	0.368863	0.457137	0.073*	
C13	0.3446 (2)	0.5742 (3)	0.2107 (3)	0.0313 (6)	
H13A	0.356492	0.490502	0.118404	0.038*	
H13B	0.409856	0.669321	0.215295	0.038*	
C14	0.4080 (2)	0.5412 (2)	0.3378 (3)	0.0305 (5)	

### Atomic displacement parameters (Å^2^)

**Table:**

	*U*^11^	*U*^22^	*U*^33^	*U*^12^	*U*^13^	*U*^23^
O1	0.0385 (9)	0.0556 (11)	0.0457 (13)	−0.0099 (8)	−0.0036 (8)	0.0315 (9)
O2	0.0435 (10)	0.0378 (9)	0.0348 (12)	−0.0124 (7)	−0.0001 (8)	0.0095 (8)
O3	0.0246 (8)	0.0319 (8)	0.0551 (13)	0.0073 (7)	0.0093 (7)	0.0217 (8)
O4	0.0259 (8)	0.0473 (10)	0.0627 (14)	0.0081 (7)	0.0063 (8)	0.0351 (9)
O5	0.0300 (8)	0.0276 (8)	0.0384 (11)	0.0054 (6)	0.0041 (7)	0.0156 (7)
O6	0.0350 (9)	0.0405 (9)	0.0339 (12)	0.0031 (7)	0.0048 (7)	0.0189 (8)
O7	0.0339 (9)	0.0460 (9)	0.0320 (12)	0.0076 (7)	0.0029 (8)	0.0159 (8)
O8	0.0354 (9)	0.0633 (11)	0.0482 (14)	0.0216 (8)	0.0067 (8)	0.0311 (10)
C1	0.0224 (10)	0.0305 (11)	0.0229 (14)	0.0026 (9)	0.0015 (9)	0.0112 (9)
C2	0.0245 (11)	0.0282 (11)	0.0243 (15)	0.0007 (9)	0.0016 (9)	0.0127 (9)
C3	0.0269 (11)	0.0283 (11)	0.0281 (15)	0.0075 (9)	0.0032 (9)	0.0144 (10)
C4	0.0219 (11)	0.0340 (12)	0.0320 (16)	0.0036 (9)	0.0019 (9)	0.0151 (10)
C5	0.0239 (11)	0.0299 (12)	0.0312 (15)	−0.0015 (9)	0.0006 (9)	0.0123 (10)
C6	0.0266 (11)	0.0283 (11)	0.0248 (15)	0.0060 (9)	0.0022 (9)	0.0118 (10)
C7	0.0233 (11)	0.0333 (12)	0.0313 (16)	0.0057 (9)	0.0019 (10)	0.0161 (11)
C8	0.0392 (14)	0.0377 (14)	0.055 (2)	−0.0118 (11)	0.0019 (13)	0.0082 (12)
C9	0.0320 (12)	0.0371 (13)	0.0486 (19)	0.0120 (10)	0.0119 (11)	0.0191 (12)
C10	0.0426 (15)	0.0641 (17)	0.060 (2)	0.0190 (13)	0.0137 (13)	0.0355 (15)
C11	0.0370 (13)	0.0257 (11)	0.0293 (16)	0.0006 (9)	0.0014 (10)	0.0111 (10)
C12	0.0506 (16)	0.0578 (17)	0.0354 (19)	0.0046 (13)	0.0034 (12)	0.0187 (13)
C13	0.0237 (11)	0.0346 (12)	0.0395 (17)	0.0064 (9)	0.0054 (10)	0.0186 (11)
C14	0.0206 (11)	0.0317 (12)	0.0389 (18)	0.0009 (9)	0.0013 (10)	0.0158 (11)

### Geometric parameters (Å, º)

**Table:**

O1—C7	1.196 (3)	C4—C5	1.374 (3)
O2—C7	1.329 (3)	C4—H4	0.9500
O2—C8	1.461 (3)	C5—C6	1.391 (3)
O3—C3	1.379 (2)	C5—H5	0.9500
O3—C9	1.429 (3)	C8—H8A	0.9800
O4—C9	1.400 (3)	C8—H8B	0.9800
O4—C10	1.425 (3)	C8—H8C	0.9800
O5—C6	1.372 (2)	C9—H9A	0.9900
O5—C11	1.429 (2)	C9—H9B	0.9900
O6—C11	1.390 (3)	C10—H10A	0.9800
O6—C12	1.417 (3)	C10—H10B	0.9800
O7—C14	1.240 (3)	C10—H10C	0.9800
O8—C14	1.308 (3)	C11—H11A	0.9900
O8—H8	0.8400	C11—H11B	0.9900
C1—C2	1.386 (3)	C12—H12A	0.9800
C1—C6	1.408 (3)	C12—H12B	0.9800
C1—C13	1.513 (3)	C12—H12C	0.9800
C2—C3	1.406 (3)	C13—C14	1.499 (3)
C2—C7	1.496 (3)	C13—H13A	0.9900
C3—C4	1.383 (3)	C13—H13B	0.9900
			
C7—O2—C8	115.27 (19)	O4—C9—O3	113.0 (2)
C3—O3—C9	116.23 (16)	O4—C9—H9A	109.0
C9—O4—C10	113.21 (19)	O3—C9—H9A	109.0
C6—O5—C11	117.44 (16)	O4—C9—H9B	109.0
C11—O6—C12	113.99 (18)	O3—C9—H9B	109.0
C14—O8—H8	109.5	H9A—C9—H9B	107.8
C2—C1—C6	119.14 (18)	O4—C10—H10A	109.5
C2—C1—C13	123.33 (18)	O4—C10—H10B	109.5
C6—C1—C13	117.51 (18)	H10A—C10—H10B	109.5
C1—C2—C3	120.27 (18)	O4—C10—H10C	109.5
C1—C2—C7	122.31 (18)	H10A—C10—H10C	109.5
C3—C2—C7	117.40 (18)	H10B—C10—H10C	109.5
O3—C3—C4	124.46 (18)	O6—C11—O5	112.89 (17)
O3—C3—C2	115.83 (17)	O6—C11—H11A	109.0
C4—C3—C2	119.71 (18)	O5—C11—H11A	109.0
C5—C4—C3	120.44 (19)	O6—C11—H11B	109.0
C5—C4—H4	119.8	O5—C11—H11B	109.0
C3—C4—H4	119.8	H11A—C11—H11B	107.8
C4—C5—C6	120.48 (18)	O6—C12—H12A	109.5
C4—C5—H5	119.8	O6—C12—H12B	109.5
C6—C5—H5	119.8	H12A—C12—H12B	109.5
O5—C6—C5	125.22 (17)	O6—C12—H12C	109.5
O5—C6—C1	114.84 (17)	H12A—C12—H12C	109.5
C5—C6—C1	119.94 (19)	H12B—C12—H12C	109.5
O1—C7—O2	122.7 (2)	C14—C13—C1	113.74 (19)
O1—C7—C2	125.0 (2)	C14—C13—H13A	108.8
O2—C7—C2	112.25 (19)	C1—C13—H13A	108.8
O2—C8—H8A	109.5	C14—C13—H13B	108.8
O2—C8—H8B	109.5	C1—C13—H13B	108.8
H8A—C8—H8B	109.5	H13A—C13—H13B	107.7
O2—C8—H8C	109.5	O7—C14—O8	123.2 (2)
H8A—C8—H8C	109.5	O7—C14—C13	122.6 (2)
H8B—C8—H8C	109.5	O8—C14—C13	114.1 (2)
			
C6—C1—C2—C3	−0.3 (3)	C13—C1—C6—O5	2.6 (3)
C13—C1—C2—C3	178.2 (2)	C2—C1—C6—C5	1.4 (3)
C6—C1—C2—C7	−178.5 (2)	C13—C1—C6—C5	−177.2 (2)
C13—C1—C2—C7	0.0 (3)	C8—O2—C7—O1	1.1 (3)
C9—O3—C3—C4	24.5 (3)	C8—O2—C7—C2	−178.97 (19)
C9—O3—C3—C2	−154.8 (2)	C1—C2—C7—O1	99.6 (3)
C1—C2—C3—O3	178.31 (19)	C3—C2—C7—O1	−78.6 (3)
C7—C2—C3—O3	−3.4 (3)	C1—C2—C7—O2	−80.3 (3)
C1—C2—C3—C4	−1.0 (3)	C3—C2—C7—O2	101.5 (2)
C7—C2—C3—C4	177.3 (2)	C10—O4—C9—O3	−67.6 (2)
O3—C3—C4—C5	−178.0 (2)	C3—O3—C9—O4	−77.8 (2)
C2—C3—C4—C5	1.2 (3)	C12—O6—C11—O5	−65.5 (2)
C3—C4—C5—C6	−0.1 (3)	C6—O5—C11—O6	−61.3 (2)
C11—O5—C6—C5	−1.9 (3)	C2—C1—C13—C14	119.8 (2)
C11—O5—C6—C1	178.44 (19)	C6—C1—C13—C14	−61.7 (3)
C4—C5—C6—O5	179.1 (2)	C1—C13—C14—O7	−29.8 (3)
C4—C5—C6—C1	−1.2 (3)	C1—C13—C14—O8	152.52 (19)
C2—C1—C6—O5	−178.9 (2)		

### Hydrogen-bond geometry (Å, º)

*Cg* is the centroid of the C1–C6 benzene ring.

**Table:**

*D*—H···*A*	*D*—H	H···*A*	*D*···*A*	*D*—H···*A*
C4—H4···O4	0.95	2.41	3.022 (3)	122
O8—H8···O7^i^	0.84	1.85	2.676 (2)	168
C9—H9*B*···O6^ii^	0.99	2.43	3.256 (3)	140
C11—H11*A*···O1^iii^	0.99	2.38	3.366 (3)	175
C13—H13*B*···O4^iv^	0.99	2.50	3.421 (3)	155
C12—H12*B*···*Cg*^v^	0.98	2.66	3.451 (3)	138

Symmetry codes: (i) −*x*+1, −*y*+1, −*z*+1; (ii) *x*, *y*+1, *z*; (iii) −*x*, −*y*+1, −*z*; (iv) *x*+1, *y*, *z*; (v) −*x*, −*y*+1, −*z*+1.
